# Breast cancer risk perceptions of Turkish women attending primary care: a cross-sectional study

**DOI:** 10.1186/s12905-014-0152-3

**Published:** 2014-12-05

**Authors:** Mehtap Kartal, Nilgun Ozcakar, Sehnaz Hatipoglu, Makbule Neslisah Tan, Azize Dilek Guldal

**Affiliations:** Department of Family Medicine, Medical Faculty of Dokuz Eylul University, 35340, Inciralti, Izmir, Turkey; Family Medicine Specialist, Ministry of Health, 24th Family Health Center, Izmir, Turkey; Family Medicine Specialist, Izmir, Turkey

**Keywords:** Breast neoplasms, Screening, Prevention, Risk, Perception

## Abstract

**Background:**

As the risks and benefits of early detection and primary prevention strategies for breast cancer are beginning to be quantified, the risk perception of women has become increasingly important as may affect their screening behaviors. This study evaluated the women’s breast cancer risk perception and their accuracy, and determined the factors that can affect their risk perception accuracy.

**Methods:**

Data was collected in a cross-sectional survey design. Questionnaire, including breast cancer risk factors, risk perceptions and screening behaviors, answered by 624 women visiting primary health care center (PHCC). “Perceived risk” investigated with numeric and verbal measures. Accuracy of risk perception was determined by women’s Gail 5-year risk scores.

**Results:**

The mean age of the participants was 59.62 ± 1.97 years. Of the women 6.7% had a first-degree relative with breast cancer, 68.9% performed breast self-examination and 62.3% had a mammography, and 82.9% expressed their breast cancer worry as “low”. The numeric measure correlated better with worry and Gail scores. Of the women 65.5% perceived their breast cancer risk accurately. Among the women in “high risk” group 65.7% underestimated, while in “average risk” group 25.4% overestimated their risk.

**Conclusions:**

Turkish women visiting PHCC are overtly and overly optimistic. This was especially obvious with the result that nearly one third had had no mammography. There is a need for further studies to understand why and how this optimism is maintained so that better screening strategies can be applied at PHCC. All health workers working at PHCC have to be aware of this optimism to prevent missed opportunities for cancer screening.

## Background

Breast cancer is by far the most frequent cancer among women with an estimation 1.383,5 million newly diagnosed cases in 2008 globally (23% of all cancers), and also is the most frequent cause of cancer death in women in both developing and developed countries [1]. In Turkey breast is most common site for cancer in women with 23.3% for women and age adjusted incidence rate is highest for breast cancer and estimated as 33.7 per hundred thousand for 2006 [2]. The mortality estimation is 13.4 per hundred thousand [3].

Despite some recent controversy, most of the current literature confirms that breast cancer screening is an effective tool for reducing breast cancer mortality [4-6]. Gaps in breast cancer knowledge, misunderstanding of related risk factors, and inaccurate perception of one’s own risk may have a negative impact on screening behaviors for breast cancer. As studies begin to quantify the risks and benefits of early detection and primary prevention strategies especially among women with high risk, the women’s risk perception of developing breast cancer has become increasingly important fact that can affect their choice of screening options or risk-reduction strategies [7-9].

Gail model, widely used as a risk-prediction method for estimating and calculating breast cancer risk, has been tested for different populations including diverse race and ethnic groups, and also validated for European women [10-12]. This model includes age of the woman, her age at menarche, and age at first live birth, number of first degree relatives with breast cancer, number of previous breast biopsies, and history of atypical hyperplasia for the estimation of breast cancer risk. Each woman’s risk can be calculated using an online calculator which generates five year and life time risk scores and the corresponding risk scores for a woman of the same age who has no risk factors [13-17].

There are conflicting results about the perceptions of women, either optimistic or pessimistic, and the effects of concern and risk perception about cancer on their screening behaviors. Women with optimistic perception, underestimating their own risk, may feel invulnerable to breast cancer and mistakenly forgo the potential benefits of screening. Moreover, if a woman is underestimating her risk but is in “high risk” group can also miss the opportunity to benefit from advances of chemoprevention in breast cancer [9,18,19]. Conversely, women with pessimistic perception, overestimating their own risk, may suffer from unnecessary worry or unnecessary evaluations that increase the chance of false-positive results leading subsequent testing or just vice versa [7,8,18,20-22].

Together with this, it is also an interesting result that the majority of women at high risk of developing breast cancer may underestimate their risk, and a substantial proportion of women at average risk may perceive that they are at increased risk [7]. But some studies have shown that the tendency of the women is leaning towards overestimating their breast cancer risk [23-25].

As the U.S. Preventive Services Task Force (USPSTF) and American Cancer Society recommend screening mammography, they consider the clinical breast exam and the breast self-examination (BSE) as a complimentary method to mammography in the context of a shared decision making and breast self-awareness of the women at average risk. Primary health care centers are the critical point of access for breast cancer screening and play a significant role in consultancy of breast health and using shared decision-making approach for cancer screening preferences of their patients [26].

The aim of the study was to evaluate the women’s breast cancer risk perception and their accuracy determined by using an objective measure, Gail model, and to investigate factors that can affect their risk perception including their socio-demographic characteristics; concerns in relation to having breast cancer, and to evaluate their screening attitudes including breast self-examination and mammography.

## Methods

Data was collected in a cross-sectional survey design that examined breast cancer risk factors, risk perceptions and screening behaviors, monthly regular breast-self examination and mammography at least once within a minimum period of five years, from cancer-free women aged over 45 years.

Women, during their visits to their primary health care center (PHCC), for any reason between March-June 2011, were invited to participate in the survey. The PHCC constitutes of 10 physicians and 11 nurses and midwives working together as a group practice. The exclusion criteria were ages below 45 and any previous personal history of breast cancer. The study was completed by 624 women having face-to-face interviews after their oral consent was taken in PHCC. Ethical approval was obtained from Dokuz Eylul University Faculty of Clinical and Laboratory Research Ethics Committee while written official permission was obtained from the Provincial Health Directorates of Izmir.

A questionnaire completed by the participants included the following: socio-demographic (age, marital status, graduation, work status) and health related characteristics (general health status, presence of first degree relatives with breast cancer, contribution of family history to breast cancer risk), risk factors included in Gail model, and screening behaviors including regular monthly BSE and mammography performance at least once within last 5 years, and breast cancer concern.

**Figure 1:**
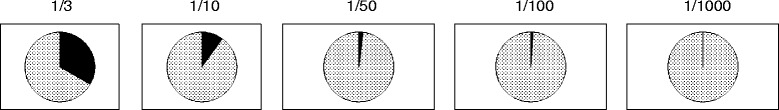
**Numeric measure for risk perceptions of women.**

“Perceived risk” was investigated with regards to numeric and verbal measures. The numeric measure was displayed with pie charts showing risk as simple fractions, risk 1 out of 3 women (1/3), 1 out of 10 women (1/10), 1 out of 50 women (1/50), 1 out of 100 women (1/100) and 1 out of 1000 women (1/1000) as shown in Figure 1.The verbal measure was asked as, “What would you say about your risk having breast cancer?”, and answered by 5-point Likert scale, namely as very low, low, moderate, high, very high. For the final analyses for risk perception, these variables dichotomized to low and high risk. Low risk included 1/100 and 1/1000 values for numeric measure and “very low, low” replies for verbal measure, while all others included in “high risk”.

Actual breast cancer risk was calculated by National Cancer Institute’s Breast Cancer Risk Assessment Tool (BCRAT), also known as the Gail Model. After entering the necessary information, calculation of the women’s breast cancer risk over the next five year was calculated with this tool available at the web site immediately [17]. The model predicts risk for breast cancer and for women whose 5-year risk scores ≥1.67 were considered as “increased-high risk” whiles others as “average risk”. This value was used as a threshold in breast cancer prevention trials [7,27,28].

Accuracy of risk perception addressed according to Gail groups, and perceived risk measures. Perceived risk categories were defined as;

- “underestimate” if women with Gail 5-year risk ≥1.67 responded as “low risk”;
- “accurate”, if women with Gail 5-year risk ≥1.67 responded as “high risk”, or women with Gail 5-year risk <1.67 responded as “low risk”; and
- “overestimate” if women with Gail 5-year risk <1.67 responded as “high risk”.

Breast cancer concern was evaluated with two items that inquired the frequency of worry and its effect on the daily life of the woman. It is scaled from 1 to 7, 1 representing “not at all” and 7 being “all the time”. Cronbach’s alpha for the study group was 0.74. The total score of the two items was grouped as low, average and high.

### Statistical analysis

Descriptive statistics were mentioned as number and percentage, while Spearman’s correlations were calculated for perceived breast cancer risks with numeric and verbal measures and objective breast cancer risk calculated with Gail model, and breast cancer concern and bivariate analysis between accuracy of perceived breast cancer risk and factors that can affect them were conducted using Pearson’s chi-square and Fisher’s exact test where appropriate using the Statistical Package for Social Sciences version 15. A two-sided alpha level was accepted as 0.05 for significance.

## Results

The age of the participants ranged from 45 to 85 years (mean age: 59.62 ± 1.97 years). About one-third of the participants (%35.4; n = 221) had graduated from primary school, most of them were not working (92.8%; n = 579), and about two-third were married/living with partner (60.6%; n = 378). Most of the women described their health as fair or good (89.3%; n = 557). Of the women, 6.7% (n = 42) had a first-degree relative with breast cancer, 52.9% (n = 330) defined the “contribution of family history to risk” as strong. Women who performed regular monthly BSE and had a mammography at least once within last 5 year constituted 68.9% (n = 430) and 62.3% (n = 389) of the participants, respectively. Most of the women, (82.9%; n = 517) expressed their breast cancer worry as “low” (Table 1). According to Gail score for 5 year breast cancer risk 22.4% (n = 140) of the women were in “increased-high risk” group with a median risk score of 2.00 (Range: 1.70-4.20) and 77.6% (n = 484) were in “average risk” group with median Gail score 1.20 (Range: 0.50-1.60).

**Table 1:** **Sociodemographic and health related characteristics of women**

	**n**	**%**
Age groups		
45-54	203	32.5
55-64	237	38.0
≥65	184	29.5
Education		
Illiterate	18	2.9
Primary school	221	35.4
Secondary school	91	14.6
High school	181	29.0
University and above	113	18.1
Work status		
Working	45	7.2
Not working	579	92.8
Marital status		
Single	32	5.1
Married/living with partner	378	60.6
Seperated/Divorced	63	10.1
Widowed	151	24.2
Health status		
Poor	22	3.5
Fair	277	44.4
Good	280	44.9
Excellent	45	7.2
First-degree relative with breast cancer		
Yes	42	6.7
No	582	93.3
Contribution of family history to risk		
Strong	330	52.9
Moderate	193	30.9
Weak	101	16.2
Regular breast self-examination		
Yes	430	68.9
No	194	31.1
Mammography within last 5 years		
Yes	389	62.3
No	235	37.7
Breast cancer worry		
Low	517	82.9
Average	59	9.5
High	48	7.7
Gail score for 5 year breast cancer risk		
Average risk (<1.67)	484	77.6
Increased-high risk (≥1.67)	140	22.4

No differences were found between “increased-high risk” and “average risk” groups according to their health status, presence of first-degree relative with breast cancer, contribution of family history to risk assessment, screening behaviors including regular BSE and mammography performance at least once within last 5 years, and breast cancer worry level of the women participated (p > 0.05).

**Figure 2:**
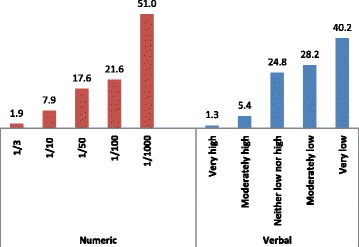
**Numeric and verbal measures of breast cancer risk perceptions of women (%).**

Breast cancer risk perceptions of the women were assessed by a numeric and verbal measures. The lowest risk value of numeric measure, “1/1000”, was mentioned by the 51.0% (n = 318) of the women as their perceived risk while 40.2% (n = 251) mentioned their risk as “very low” in verbal measure (Figure 2).

Accuracy of risk perception determination was done by using numeric measure as it had stronger correlations with worry and Gail scores (Table 2).

**Table 2:** **Correlations of perceived and estimated breast cancer risk and worry of women**

	**Numeric**	**Verbal**	**Worry**
Numeric measure	-		
Verbal measure	0.622**	-	
Breast cancer worry	0.328**	0.254**	-
Gail score	0.117**	0.083*	-0.011

*p < 0.05.

**p < 0.001.

**Figure 3:**
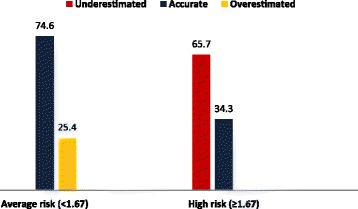
**Distribution of accuracy of risk perceptions of women according to Gail 5-year risk scores (%).**

Most of the women, 65.5% (n = 409) perceived their breast cancer risk accurately. Among the women in “increased-high risk” group according to Gail score for 5 year breast cancer risk 65.7% (n = 92) underestimated their risk, while 25.4% (n = 123) of women in “average risk” group overestimated their risk (Figure 3).

Women aged between 45-54 years estimated their risk as “high” more than when compared to the older age groups (p < 0.001). Women who had an education level equal to a university degree and above and who worked overestimated their risk more than the other groups (p = 0.019 and p = 0.015, respectively). Higher proportion (34.4%) of the single women underestimated their risk (p = 0.001). The accuracy of breast cancer risk estimation was not affected by presence of a first-degree relative with breast cancer, health status of the women, monthly BSE and mammography performance at least once within last 5 years (p > 0.05). Women who described the “contribution of family history to risk” as weak were statistically inclined to significantly less overestimate their risk (p = 0.008). Women with “low” breast cancer worry have less overestimated their risk than other groups (p < 0.001) (Table 3).

**Table 3:** **Distribution of accuracy of risk perceptions of women according to sociodemographic and health related characteristics**

		**Underestimated**		**Accurate**		**Overestimated**		**p-value**
		**n**	**%**	**n**	**%**	**n**	**%**	
Age groups	45-54	8	3.9	131	64.5	64	31.5	<0.001
	55-64	35	14.8	157	66.2	45	19.0	
	≥65	49	26.6	121	65.8	14	7.6	
Education	Illiterate	4	22.2	14	77.8	0	0.0	0.019
	Primary school	25	11.3	153	69.2	43	19.5	
	Secondary school	18	19.8	55	60.4	18	19.8	
	High school	22	12.2	127	70.2	32	17.7	
	University and above	23	20.4	60	53.1	30	26.5	
Work status	Working	1	2.2	30	66.7	14	31.1	0.015
	Not working	91	15.7	379	65.5	109	18.8	
Marital status	Single	11	34.4	20	62.5	1	3.1	0.001
	Married/partner	50	13.2	239	63.2	89	23.5	
	Divorced	7	11.1	41	65.1	15	23.8	
	Widowed	24	15.9	109	72.2	18	11.9	
Health status	Poor	4	18.2	13	59.1	5	22.7	0.351
	Fair	41	14.8	170	61.4	66	23.8	
	Good	41	14.6	194	69.3	45	16.1	
	Excellent	6	13.3	32	71.1	7	15.6	
1st-degree relative with breast cancer	Yes	7	16.7	25	59.5	10	23.8	0.689
	No	85	14.6	384	66.0	113	19.4	
Contribution of family history to risk	Strong	44	13.3	222	67.3	64	19.4	0.008
	Moderate	24	12.4	121	62.7	48	24.9	
	Weak	24	23.8	66	65.3	11	10.9	
Breast self-examination	Yes	62	14.4	288	67.0	80	18.6	0.499
	No	30	15.5	121	62.4	43	22.2	
Mammography within last 5 years	Yes	58	14.9	252	64.8	79	20.3	0.862
	No	34	14.5	157	66.8	44	18.7	
Breast cancer worry	Low	83	16.1	356	68.9	78	15.1	<0.001
	Average	8	13.6	25	42.4	26	44.1	
	High	1	2.1	28	58.3	19	39.6	

## Discussion

The purpose of the study was to determine the breast cancer risk perception in women and their accuracy according to the Gail model, and to examine factors that can affect their risk perception. The results revealed that, although most of the Turkish women visiting PHCC perceived their breast cancer risk accurately, the ones determined as “increased risk” were more likely to be optimistic. The women working, having higher education levels and aged between 45-54 years were overestimating their risk.

Different measures were used for risk perception including numeric, verbal, and comparative. The accuracy of risk perception is an issue that can be affected by the measure used. It was shown that numerical measure of absolute risk was more highly correlated than measures of comparative risks [24]. In this study, the numeric measure was also found to be better than verbal measure as it had a better correlation with Gail scores of the women so it was used as the accuracy measurement of risk perception of the women.

The current literature shows that women have difficulty in estimating their breast cancer risk accurately with regards to different measures used in different forms [7,20-23,29]. This is also true for women working as health professionals [30]. Two studies used the number 1.67 as the cut off point for Gail 5 year risk score in relation to average and increased-high risk in comparison of perceived risk. Haas and et al. showed that among women with different race and ethnicity in average risk group with a median Gail score 1.00 (range = 0.32-1.66) 28% overestimate their breast cancer risk while in increased-high risk with median Gail score 2.21 (range = 1.67-12.13) 56.9% underestimate their risk [7]. Banegas and et al. states that for Chilean women, who are among the women in the “average risk” group, in accordance with the BCRAT 5 year risk of breast cancer 13.7-22.5% overestimate whilst the “increased-high risk” group showed to underestimate by 52.6-57.9% their breast cancer risk with absolute and comparative measures for perceived risk, respectively [11]. Another study indicated that ≤9% of low-average-risk women overestimated their breast cancer risk, whereas ≥80% of increased-high-risk women underestimated their breast cancer risk [18]. In our study, according to Gail score for 5 year breast cancer risk, women in “average risk” group overestimated their risk in similar percentages however women in “increased-high risk” group were more likely to underestimate their risk showing their optimistic view. In a meta-analysis it was found that the studies’ sample concluding overestimation of breast cancer risk selected via a relative who had a history breast cancer or from a healthcare setting including a hospital, a primary care, or a genetic counseling clinic. By contrast, studies that reported an optimistic bias for the perceived risk had their samples from the community [31]. Our sample was recruited from a PHCC, and although it is a healthcare setting, our results showed the optimistic bias of the samples from the community. It is not a surprising outcome as PHCCs are the best settings that reflect our community. It seems that primary care workers including physicians, nurses and midwives have to face with the reality of the handicaps of this optimistic view of the women they meet in their daily practice.

It is obvious that women’s socio-demographic characteristics can have an impact on their risk perception. There are pros and cons in the results of studies for younger women’s risk perception for breast cancer. While most of the studies reported younger ages were inversely associated with risk perception or in other words to overestimate their risk like we found [7,9,11,18,20,32,33], the two studies from Turkey reported just the opposite [30,34]. However, a meta-analysis overall found no relationship between older age and increased perceived risk [31]. There are also controversial results for risk perception accuracy of women according to their education, marital status, and income [9,20,29,33]. The study population of women who were highly educated, working, married or divorced tended to show a pessimistic perception about their breast cancer risk. Researchers in their meta-analysis found that women with college education were less likely to have an optimistic bias [31].

Presence of a first-degree relative with breast cancer and the concern levels of women are the other important issues that can have affect on screening behaviors of the women. In different study populations, the percentage of first-degree relative with breast cancer changes between 2.9% and 11% [9,11,28-30]. The percentage of women performing BSE ranges between 21.3-83.7, while the percentage of women having mammography at least once changes between 20.9-78.9 [9,30,34-37]. In our study population, 6.7% of the women had a first-degree relative with breast cancer, and nearly one third of women did not perform regular monthly BSE or have a mammography at least once within last 5 years. Although high levels of cancer worry are uncommon, as found in our study, higher worry levels are not associated with reduced screening. Most of the studies reported that breast cancer worry, regardless of how it is measured, is associated with stronger likelihood of screening, mammography or BSE. However, there were exceptions including a study population of women who just had had a biopsy to determine whether they had breast cancer or not and one that found no relation between worry and BSE where 21.3% of women perform BSE regularly once a month [34,38].

All studies showed that women who had risk factors for breast cancer such as previous personal history of breast disease, presence of family history of breast cancer, and being of an age over 40, having their first menstrual period at a young age, history of abnormal mammography, history of breast biopsy or morbid obesity were more likely to rate their perceived risk as “moderate-strong” or “increased” [7,9,30-32,36]. In our study women who had family history of breast cancer had a pessimistic perception. However, women who described the “contribution of family history to risk” as weak significantly underestimated their risk. The knowledge gap about a risk factor for breast cancer, like presence of family history of breast cancer can be a factor that effects the risk perception.

There are also conflicting results reported about the perceived risk and screening behaviors of women including adherence to BSE and mammography. Studies showed that the increased perceived cancer risk was related to increased regular breast cancer screening or mammography use [30,32,37]. However, even among the women with the highest projected breast cancer risk, approximately 25% did not report routine mammography use. On the other side, increased 5-year projected breast cancer risk was associated with a statistically significant increase in recent or routine mammography use [32,36]. The women who had had a mammogram in the last year showed both accurate and high risk perception [9,29]. However, we could not find any relationship between risk perception and regular monthly BSE or mammography performance. Katapodi et al. showed that perceived risk has a small but significant effect on mammography adherence for cancer screening. However, there were inconclusive results about the association between BSE adherence and perceived risk [31].

Greater worries about breast cancer were found to be associated with higher perceived verbal/numerical absolute risk [24]. In our study, most of the women expressed their breast cancer concern as “low”, and among them those who described their worry as “low” were more optimistic than others. Women with optimistic view might feel invulnerable to breast cancer and screening guidelines [19].

To our knowledge, this is one of few studies which investigated the risk perception of Turkish women, and factors that can have effect on the perception of risk. However, the study has some limitations. As it has the design of a cross-sectional study, a causal relationship cannot be identified. Secondly, all study participants were recruited from a PHCC which means the results may not be generalizable to their peers in Turkey. Finally, there may have been some information bias in terms of the risk perception and worry levels because these variables were measured using self-reported questionnaires.

## Conclusions

In conclusion, the current study revealed that, Turkish women, especially those in the “increased risk” group, visiting the PHCCs were more likely to underestimate their risk for breast cancer and it was found that nearly one third did not have a mammography at least once within last 5 years. Not only physicians, but also nurses and midwives working at PHCCs have to be aware of this optimism in order to prevent missed opportunities of diagnosis while consulting and managing their cancer screening. There is also a need for further studies that aim to understand why and how this optimism is maintained so that better screening strategies can be applied at PHCCs.

## Abbreviations

PHCC: Primary health care center
USPSTF: U.S. Preventive Services Task Force
BSE: Breast self-exam
BCRAT: Breast Cancer Risk Assessment Tool
